# Retrograde Type A Aortic Dissection 48 Hours after TEVAR in a Patient with a Delayed Diagnosis of Vascular Ehlers-Danlos Syndrome

**DOI:** 10.1055/s-0042-1749171

**Published:** 2022-11-01

**Authors:** Ian Diebels, Jeroen M.H. Hendriks, Joke Durnez, Bernard P. Paelinck, Haroun El Addouli, Steven Laga, Bart L. Loeys

**Affiliations:** 1 Department of Thoracic and Vascular Surgery, Antwerp University Hospital, Edegem, Belgium; 2 European Reference Network Qualified Aorta Clinic of the Antwerp University Hospital, Antwerp University Hospital, Edegem, Belgium; 3 Department of Cardiology, Antwerp University Hospital, Edegem, Belgium; 4 Department of Radiology, Antwerp University Hospital, Edegem, Belgium; 5 Department of Cardiac Surgery, Antwerp University Hospital, Edegem, Belgium; 6 Department of Genetic Medicine, Antwerp University Hospital, Edegem, Belgium

**Keywords:** acute aortic syndrome, aortic dissection, thoracic endovascular aortic repair, vascular Ehlers-Danlos syndrome, connective tissue disorder

## Abstract

We report a case of a fatal retrograde Type A aortic dissection following thoracic endovascular aortic repair (TEVAR). The patient was diagnosed with vascular Ehlers-Danlos syndrome (vEDS) only postoperatively, which is a relative contraindication for TEVAR. The patient had no major or minor criteria for vEDS. This case report emphasizes pitfalls of TEVAR in patients with a connective tissue disorder.

## Introduction

Aortic dissection (AoD) is characterized by a sudden separation of the aortic wall, leading to the formation of a true and a false lumen. For complicated Stanford Type B AoD, thoracic endovascular aortic repair (TEVAR) is the treatment of choice. 1 However, there are several important complications that may occur, including retrograde Type A AoD (RTAoD).

When treating AoD, it is important to exclude connective tissue disorders, including Marfan syndrome, Ehlers-Danlos syndrome (EDS), and Loeys-Dietz syndrome, since open surgical repair is favored over endovascular repair in nonurgent settings. 2

In this article, we present the fatal case of a Stanford Type B AoD complicated by a RTAoD after TEVAR in a patient without any criteria for vascular EDS (vEDS) preoperatively, but in whom a postoperative molecular diagnosis of vEDS was made.

## Case Presentation

**Fig. 1:**
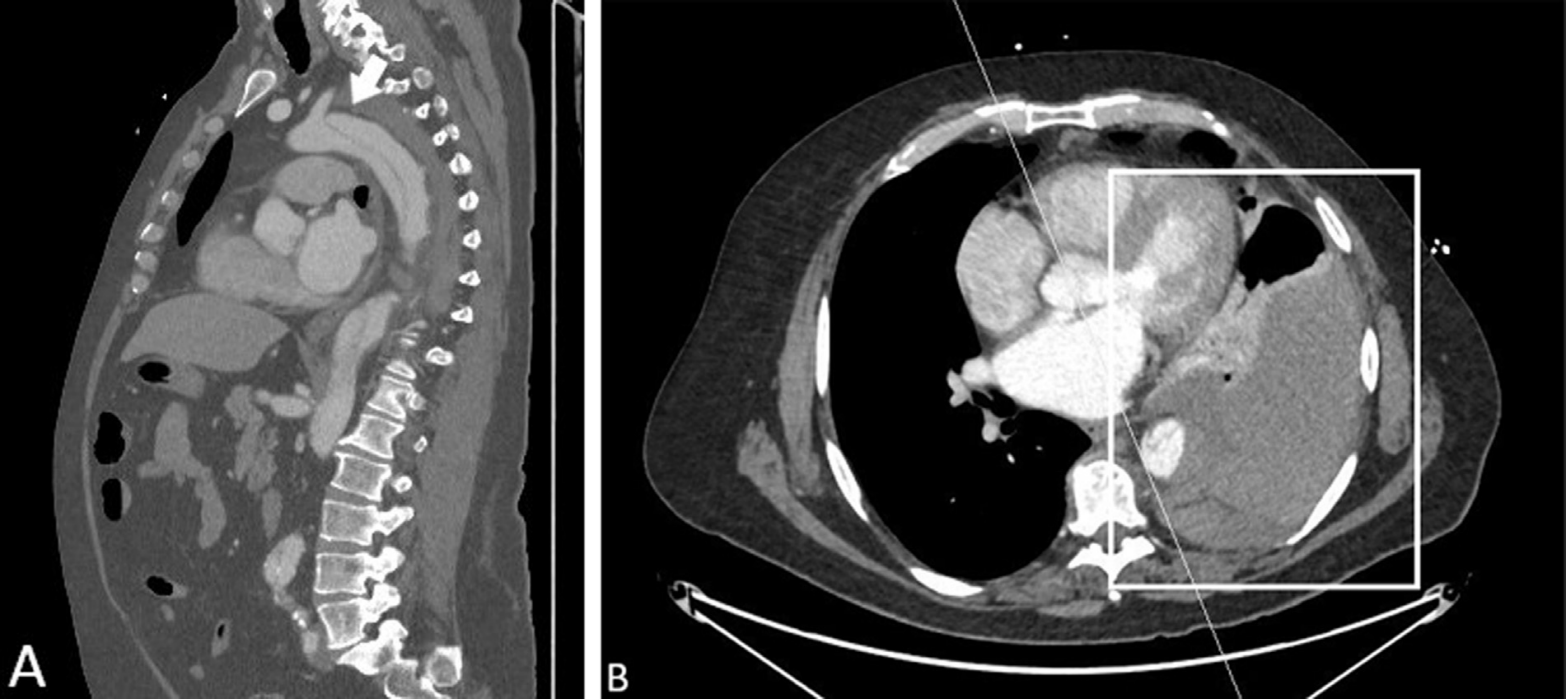
Computed tomography angiography images at initial presentation. ( **A** ) Stanford Type B aortic dissection, originating distal to the left subclavian artery. Arrows mark the false lumen. ( **B** ) Left-sided hemothorax (box).

**Fig. 2:**
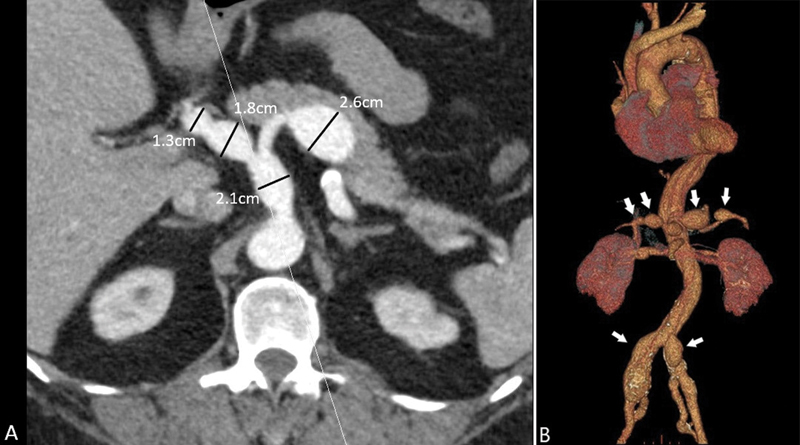
Computed tomography angiography images showing multiple artery aneurysms: ( **A** ) celiac trunk (2.6 and 2.1 cm), hepatic artery (1.8 cm), and left gastric artery (1.3 cm). ( **B** ) Computed tomography angiography three-dimensional reconstruction with arrows marking the aneurysms.

A 58-year-old male presented with acute chest pain radiating toward the interscapular region. A computed tomography (CT) scan revealed a Stanford Type B, DeBakey Type III, AoD. The AoD originated near the left subclavian artery and extended down to the iliac vessels ( Fig. 1 ). CT also showed a left-sided hemothorax, confirmed by the average density of 40 to 55 HU ( Fig. 1 ), and aneurysms in the visceral and iliac arteries ( Fig. 2 ).

His past medical history was uneventful.

The patient was hemodynamically stable. He was admitted to the intensive care unit for optimal medical treatment. The patient's pain was not brought under control by medical therapy, although he remained normotensive while receiving bisoprolol (a beta-blocker) and urapidil (an alpha-blocker).

In combination with the earlier identified left-sided hemothorax, it was felt an intervention was warranted.

We performed TEVAR using two Medtronic Valiant thoracic stent-grafts with a maximum 10% diameter oversizing. Both endoprostheses were deployed during a short period of controlled hypotension. Angiography showed correct positioning of the endoprostheses, with a patent left subclavian artery. We did not perform postdilation with a balloon catheter, and the procedure was performed in our angio suite with the Artis ZEEGO angiography system (Siemens Healthcare, Erlangen, Germany).

**Fig. 3:**
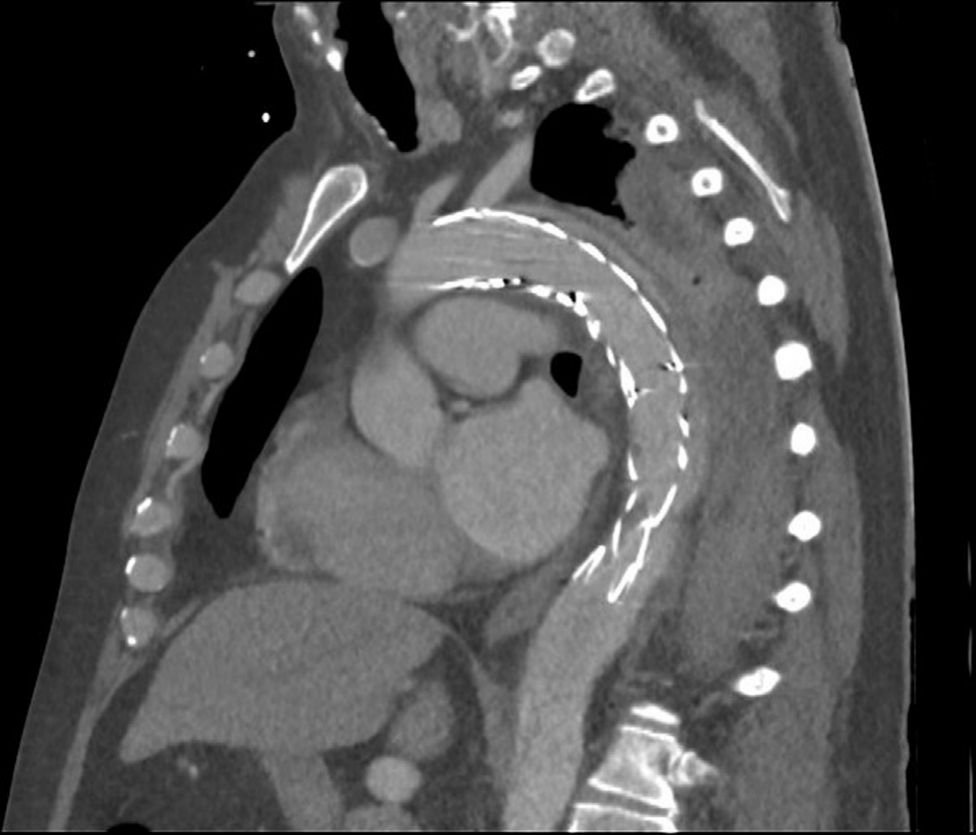
Computed tomography angiography showing a correctly placed aortic endoprosthesis in the absence of complications.

On the second postoperative day, an elective follow-up CT angiography was performed, confirming correct positioning of the aortic endoprostheses ( Fig. 3 ).

However, the patient expressed severe retrosternal pain again. Urgent transthoracic echocardiography showed cardiac massive tamponade, which rapidly deteriorated into cardiac arrest.

While receiving cardiopulmonary resuscitation, the patient was transferred to the operating theater for immediate open surgical repair. Suspected retrograde dissection from the endoprosthesis with rupture of the ascending aorta was confirmed. An aortic arch replacement with Dacron and a supracoronary root repair with polytetrafluoroethylene were performed under deep hypothermic circulatory arrest with selective antegrade cerebral perfusion. Aortic biopsies were obtained and sent for histopathological examination and genetic analysis.

A transesophageal echocardiography on day 1 postoperatively showed only a trace of aortic insufficiency. The patient deteriorated during his postoperative stay in the intensive care unit. A head CT scan showed diffuse cerebral edema and impending brain herniation, and the patient passed away shortly afterwards.

Postmortem analysis via a thoracic aortic aneurysm and dissection-panel genetic screening (including 34 genes) revealed a heterozygote c.1024G > A p.(Gly342Arg) pathogenic variant in exon 15 in *COL3A1* , confirming the molecular diagnosis of vEDS.

## Discussion

Vascular Ehlers-Danlos is a rare autosomal dominant connective tissue syndrome caused by a heterozygous mutation in *COL3A1* , which results in reduced or defective production of Type III collagen. It is estimated that approximately 1 in 50,000 to 250,000 people are affected by the vascular type of Ehlers-Danlos (vEDS). 2 3 The prognosis of vEDS is poor, with a median life expectancy of 40 to 50 years. Approximately 7% of patients with vEDS experience a first major event by the age of 20 years, which further increases to 40% by the age of 40 years. 3

The 2017 Revised International Classification of Ehlers-Danlos Syndromes identified five major criteria for the clinical diagnosis of vEDS: (1) a family history of vEDS with a documented causative variant in *COL3A1* , (2) arterial rupture or dissection under the age of 40 years, (3) spontaneous sigmoid colon perforation, (4) spontaneous uterine rupture during the third trimester, and (5) spontaneous carotid-cavernous sinus fistula formation. Twelve minor clinical criteria have also been identified, which include typical facial, body, and skin features, among others. The presence of a major criterion or multiple minor criteria warrants further diagnostic testing, such as biochemical analysis of Type III procollagen and/or direct deoxyribonucleic acid analysis of the *COL3A1* gene. 2 4 Sequencing analysis of *COL3A1* has a high sensitivity (98%) to identify the underlying pathogenic variant. However, if vEDS is suspected but no *COL3A1* mutation can be identified upon routine sequencing analysis, whole exome or genome sequence analysis or analysis of messenger ribonucleic acid from cultured fibroblasts may identify pathogenic variants. 2 Unfortunately, these analyses take several weeks to conclude and are therefore not available in the acute setting.

None of the above-mentioned criteria were present in our patient. The patient had a family history of sudden cardiac death in his father at age 62 years (no autopsy performed). Also a paternal uncle died at age 45 due to AoD.

Our case illustrates that treatment of vascular events should be as conservative as possible in case vEDS is diagnosed or suspected. 2 3 Due to the fragility of the blood vessels, many frequently used surgical or endovascular techniques can damage the vessels even more, possibly resulting in larger tears and/or ruptures. 2

Data on aortic stent grafting (endovascular aneurysm repair [EVAR], TEVAR) for patients with vEDS are limited, and recommendations of EVAR and TEVAR are often extrapolated from pooled connective tissue disorder studies and Marfan's syndrome in particular. Many complications of aortic stent graft placement appear to result from direct injury to the intima from the stent graft, inducing a so-called “stent graft-induced new entry” (SINE) tear. 5 In EDS, the mortality rates for open and endovascular repair range from 20 to 30%. 3 6 It is generally advised that endoprostheses should be reserved for acute life-saving interventions, late chronic pseudoaneurysms, graft-to-graft approach, following open aortic arch replacement, or in cases with significant risk for open surgical approach. 3 7

Also, due to the risk of pseudoaneurysm formation, a surgical cutdown with pledgeted direct suture of the access vessel, rather than percutaneous approach, should be considered in vEDS patients. 8 If a percutaneous approach is chosen, duplex-guided punctures are essential. Also the use of devices with proximal uncovered struts should be avoided, as they might induce new entry tears leading to RTAoD in up to 25% of cases. 3 7 8 In general, endograft oversizing of more than 10% and the use of a molding balloon are contraindicated in every AoD patient, as these may further damage the weakened aortic wall. 6 8 The role of bare distal stents (composite device design) remains undefined. Finally, endoprosthesis placement at the thoracic level should be done under permanent invasive blood pressure monitoring, with brief blood pressure reduction or rapid ventricular pacing at the time of deployment, to prevent distal stent migration. 1 2
